# Detection of Bovine Leukemia Virus in Brains of Cattle with a Neurological Syndrome: Pathological and Molecular Studies

**DOI:** 10.1155/2013/425646

**Published:** 2013-04-28

**Authors:** Rubens Henrique Ramos D'Angelino, Edviges Maristela Pituco, Eliana Monteforte Cassaro Villalobos, Ricardo Harakava, Fábio Gregori, Claudia Del Fava

**Affiliations:** ^1^Pós-Graduação em Sanidade, Segurança Alimentar e Ambiental no Agronegócio, Instituto Biológico de São Paulo (IB), Avenida Conselheiro Rodrigues Alves 1252, Bairro Vila Mariana, 04014-002 São Paulo, SP, Brazil; ^2^Laboratório de Viroses de Bovídeos, Centro de P & D de Sanidade Animal (CPDSA), IB, Avenida Conselheiro Rodrigues Alves 1252, Bairro Vila Mariana, 04014-002 São Paulo, SP, Brazil; ^3^Laboratório de Raiva e Encefalites, CPDSA, IB, Avenida Conselheiro Rodrigues Alves 1252, Bairro Vila Mariana, 04014-002 São Paulo, SP, Brazil; ^4^Laboratório de Bioquímica Fitopatológica, Centro de Sanidade Vegetal, IB, Avenida Conselheiro Rodrigues Alves 1252, Bairro Vila Mariana, 04014-002 São Paulo, SP, Brazil; ^5^Laboratório de Biologia Molecular e Sorologia Aplicadas, Departamento de Medicina Veterinária Preventiva e Saúde Animal, VPS, Faculdade de Medicina Veterinária e Zootecnia da Universidade de São Paulo, Avenida Professor Dr. Orlando Marques de Paiva 87, Cidade Universitária, 05508-270 São Paulo, SP, Brazil; ^6^Laboratório de Anatomia Patológica, CPDSA, IB, Avenida Conselheiro Rodrigues Alves 1252, Bairro Vila Mariana, 04014-002 São Paulo, SP, Brazil

## Abstract

Bovine leukemia virus (BLV) was investigated in the central nervous system (CNS) of cattle with neurological syndrome. A total of 269 CNS samples were submitted to nested-PCR (BLV env gene gp51), and the viral genotypes were identified. The nested-PCR was positive in 4.8% (13/269) CNS samples, with 2.7% (2/74) presenting at histological examination lesions of nonpurulent meningoencephalitis (NPME), whereas 5.6% (11/195) not presenting NPME (*P* > 0.05). No samples presented lymphosarcoma. The PCR products (437 bp) were sequenced and submitted to phylogenetic analysis by neighbor-joining and maximum composite likelihood methods, and genotypes 1, 5, and 6 were detected, corroborating other South American studies. The genotype 6 barely described in Brazil and Argentina was more frequently detected in this study. The identity matrices showed maximum similarity (100%) among some samples of this study and one from Argentina (FJ808582), recovered from GenBank. There was no association among the genotypes and NPME lesions.

## 1. Introduction

Bovine encephalitis is an important group of usually fatal diseases that have a strong impact on public health, result in major economic losses worldwide, and present a sanitary barrier to international trade [1]. Agents associated with these diseases include viral, bacterial, parasitic, neoplastic, toxic, and metabolic agents, and all must be considered during differential diagnosis [2].

The importance of differential diagnosis of neurologic syndromes in cattle has increased since 1985, when bovine spongiform encephalopathy (BSE) was first identified in the United Kingdom [3]. Its association with the emergence of a new variant of the human disease Creutzfeldt-Jakob Disease (CJD) increased its political, social, and public health significance. International health authorities require evidence from countries exporting meat, which includes Brazil, that their herds are free from BSE and that the causes of encephalitis and encephalopathy are diagnosed and clarified [4]. Decree no. 516 dated December 9, 1997, introduced a BSE surveillance component into the system of surveillance of Herbivore rabies as part of the National Rabies Control of herbivores (PNCRH) of the *Ministério da Agricultura, Pecuária e Abastecimento* (Ministry of Agriculture, Livestock and Supply, MAPA) [5]. Thus, animals with symptoms of central nerve system disorders must be submitted to a differential diagnosis for rabies and other encephalitides and encephalopathies. These rules are intended to improve specific epidemiological surveillance measures to ensure a country free of BSE [6]. The differential diagnosis and prevention of BSE and the livestock assets in Brazil is of fundamental importance, as is evaluating the risks related to public health and the economic outlook. 

Analysis conducted by the Biological Institute from April to July 2002, of samples from 131 cattle with a CNS neurological syndrome [1], confirmed the etiologic agent in only 38.9% of the samples. Also 21.1% were positive for rabies (direct immunofluorescence and biological tests), 1.5% positive for Bovine Viral Diarrhea Virus (BVDV) (isolation, identification by immunoperoxidase and RT-PCR), 0.7% positive for BoHV-5 (PCR), and 0.7% were positive for *Neospora caninum* (conventional PCR). In 16.0% of the cases, isolation and identification of bacteria (*Listeria monocytogenes*, *Streptococcus* spp., *Clostridium perfringens*, *Arcanobacterium pyogenes*, *Enterobacter cloacae*, *Pseudomonas aeruginosa*, *Acinetobacter* spp., and *Staphylococcus* spp.) were obtained. In 61.1% of the samples, the agent could not be identified using available diagnostic methods (isolation and molecular) making it necessary to investigate other agents and employ other methods. 

The Anatomopathology Laboratory of the Biological Institute of São Paulo has been accredited by MAPA resolution number 5 of January 8, 2004 (Federal Official Gazette of Brazil of 09.01.2004, Section 1, page 3), to perform histopathologic differential diagnosis of encephalitis and BSE, thus meeting MAPA's PNCRH criteria. From January 2004 to June 2007, 690 brains of cattle with clinical signs of nervous disorders associated with rabies (direct immunofluorescence) were submitted for histologic examination, with 40% (276) showing pathological changes and nonpurulent and nonspecific meningoencephalitis being the most frequent lesions observed, at 90.0% (249/276) [7]. This type of inflammatory mononuclear infiltrate indicates microorganisms (viral agents, bacteria, and parasites) causing encephalitis, necessitating sensitive, and specific tests to determine the causative agents.

Bovine leukemia virus (BLV), which causes bovine leukosis (BL), is widespread in all regions of Brazil with high incidence of seroreactivity in dairy herds and other livestock under intensive management [8], although lymphosarcoma in the CNS has been reported as a rare condition in cattle [9, 10]. 

Del Fava et al. [10] reported the occurrence of 0.03% (1/2867) intracerebral lymphosarcoma in bovine with neurological syndrome, in a surveillance period of seven years, in Brazil. The tumoral mass was positive to nested-PCR (BLV env gene gp51). The RFLPA analyses using enzyme* Bam *HI revealed a site in amplified segments of BLV env gene, providing information about its population polymorphism, and allowed to classify into the Japanese-North American subgroup. The amplified proviral DNA was analyzed using ABI Prism 377 DNA sequencer (Applied Biosystems), and the obtained sequence was aligned and compared with others present in GenBank, by program BLAST, confirming the subgroup.

Given that BLV induces seroconversion, causes persistent lymphocytosis, and may rarely lead to lymphosarcoma, and that there are many cases with non-purulent meningoencephalitis (NPME) in which a causative diagnosis cannot be made, the aim of this study was to investigate BLV as a possible cause of neurological syndrome in cattle testing negative for rabies.

## 2. Materials and Methods

Bovine leukemia virus infection was verified from CNS samples of 269 cattle with neurological symptoms referred to the *Centro de Pesquisa e Desenvolvimento de Sanidade Animal of Instituto Biológico* (Center of Research and Development of Animal Health of the Biological Institute) from January 2007 to December 2009, for differential diagnosis of neurological syndrome. The samples were collected by government and private veterinarians, and all samples were negative for rabies virus based on the antirabies conjugate labeled with fluorescein isothiocyanate (Sanofi ) and *in vivo* testing [11].

Tissues were fixed in 10% buffered formalin, cut into smaller pieces, and then dehydrated, cleared, and embedded in paraffin; sections were cut at 5 *μ*m and stained with hematoxylin and eosin [12].

DNA extraction was made from chilled tissue samples using the Wizard *Genomic* DNA *Purification* kit (Promega Corporation, Madison, WI, USA—Cat. no. A1120). Amplification of the segment that encodes the gp51 env gene of the BVL was conducted by nested PCR, using specific primers for amplifying a segment of 440 base pairs (bp) [13]. Briefly, the PCR reaction conditions were as follows: volume of DNA in the first and second amplification was 5 *μ*L and 1.5 *μ*L, respectively; concentration of external and internal primers, 0.2 and 0.1 *μ*M; initial denaturation at 94°C for 2 min; 40 repeat cycles of denaturation at 95°C for 30 sec; annealing at 62°C for 30 sec in the first and 70°C for 30 sec in the second amplification; extension at 72°C for 1 min; final extension at 72°C for 4 min. Analysis of amplified products was by electrophoresis (100 V/60 min) agarose gel at 1.5% in Tris plug, sodium acetate, EDTA pH 8.0, and stained with ethidium bromide. The image of the gel under UV light was recorded using camera coupled to a computer.

The positive PCR products were purified with the Wizard Genomic DNA Purification kit (Promega Corporation, Madison, WI, USA—Cat. no. A7170) and subjected to sequencing reaction by chain termination with dideoxynucleotides marked with fluorophores. The reaction was performed with 5.68 *μ*L of the PCR product, 2 *μ*L reagent Big Dye 3.1 (Applied Biosystems), 2 *μ*L of dilution buffer (0.2 M Tris-HCl pH 9.0, 5 *μ*L MgCl_2_), and 0.32 *μ*L of a primer used in PCR (final concentration 0.32 *μ*L) to a total volume of 10 *μ*L. Each sample was sequenced in both directions using forward and reverse primers. The samples were incubated in a thermocycler (PTC-100, MJ Research) for 25 cycles at 95°C for 10 sec and 60°C for 4 min. The sequencing reaction products were precipitated by adding 40 *μ*L of 75% isopropanol and centrifugation R-5810 centrifuge (Eppendorf) at 3220 ×g for 30 min at room temperature. After discarding the supernatant, another 100 *μ*L of 75% isopropanol was added for washing the precipitate, and the supernatant was again discarded, without centrifugation. The precipitate was dried by incubation at 37°C for 30–60 min. The precipitate was re-suspended in 2 *μ*L of formamide, denatured at 95°C for 2 min, and subjected to electrophoresis in denaturing polyacrylamide gel in ABl377 automated sequencer (Applied Biosystems).

The generated sequences were analyzed by the program Bioedit v.7.0.9 [14] to generate a unique sequence from the bidirectional sequence data. They were then aligned to each other and to homologues retrieved from Genbank, using the Clustal W version 1.8.3 software [14].

After definition of a consensus block, a table of nucleotide and amino acid identity was constructed using the *software* Bioedit v.7.0.9 [14]. Phylogenetic inference was performed using the program Mega, version 5 [15]. The tree generated from the nucleotide sequence was constructed with the neighbor-joining method using the substitution pattern *maximum composite likelihood* for the partial region (437 nt) of the env glycoprotein 51 gene from the BLV (nt 5110–5546 having as reference a sample of BLV, Argentina Accession no. AF257515), representing approximately 50% of the total gene, with the bootstrap values defined from 1000 replicates, with values equal to or greater than 50 submitted to the next node.

The association between the histopathologic findings of non-purulent, nonspecific meningoencephalitis and results of nested PCR, positive or not, was evaluated using Fisher's two-tailed exact test with an alpha error 5% [16].

## 3. Results and Discussion

The 269 CNS tissue samples from cattle with a CNS neurological syndrome showed a high number with characteristic lesions of NPME, corroborating with Del Fava et al. [7], but the nested-PCR showed low positivity for BLV (Table 1). These data indicated that other infectious agents, including rabies, are important in the differential diagnosis of diseases of the CNS in cattle, as was demonstrated in southern Brazil [4].

Statistical analysis by Fisher's exact test [16] showed no significant (*α* = 5%, *P* = 0.52, CI = 0.1073 to 2.083) positive correlation between nested-PCR results and the presence of lesions characteristic of NPME.

Taking into account the high seroprevalence of BLV in recent decades in Brazil [17–19], especially in dairy herds, it is suspected that the low detection of genetic material (proviral DNA) of BLV by nested-PCR (13/269, 4.8%) may be due to low or absent neurotropism and/or in the difficulty of the virus in crossing the blood-brain barrier, even in animals with inflammatory changes of the CNS. The number of cases found positive with nested-PCR in samples with NPME (15.4%—2/13) was low in the present study. Another possible reason for this discrepancy is the connection of BLV with lymphoid tissue, particularly B lymphocytes [20, 21], and not the CNS itself. For this reason, a higher incidence of neoplastic changes is found in lymph nodes than in spleen, heart, uterus, abomasum, liver, and kidneys [22].

Positivity to BLV in animals with NPME may be due to the presence of the integrated virus in the lymphocyte genome, which is infiltrated in the tissues. On the other hand, in the positive nested-PCR animals that did not reveal NPME (84.6%—11/13), it can be assumed that the presence of proviral DNA in some samples may have been due to amplification of the genetic material from the intraluminal lymphocytes of cerebral blood vessels, taking into account that around 30%–70% of infected animals have shown persistent lymphocytosis [23].

**Figure 1:**
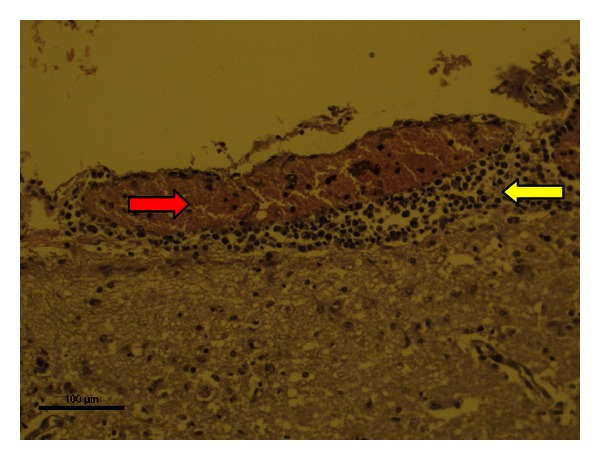
Nonpurulent meningitis (H & E 200x magnification). Bovine brain, positive for BLV, with accumulation of mononuclear inflammatory cells along the meninges (yellow arrows) and apparent congestion (red arrow).

**Figure 2:**
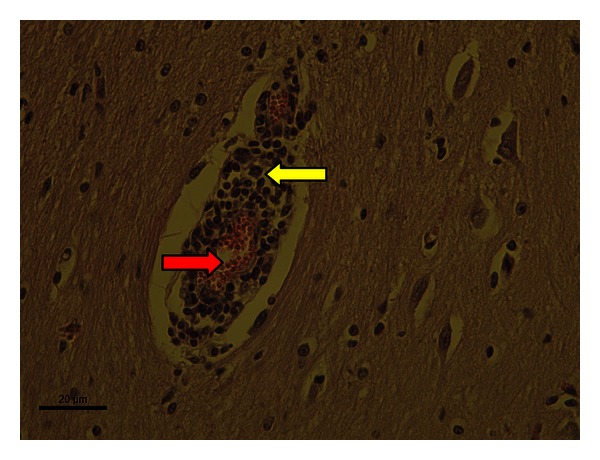
Non-purulent encephalitis (H & E 400x magnification). Bovine brain, positive for BLV, with mononuclear cell infiltrates in the Virchow-Robin space (yellow arrows) characterizing the perivascular cuffing. The red arrows indicate the vascular lumen, with red blood cells within.

In samples showing characteristics consistent with NPME, regardless of BLV status, meningeal inflammatory infiltration (Figure 1) and accumulation of inflammatory cells along the Virchow-Robin space were evident (perivascular cuffing) (Figure 2).

Comparison of the nucleotide and amino acid identity matrix (Table 2) of the sequenced samples from this work showed 100% identity among the BLV samples JN254633, JN254637, and JN254638, all from São Paulo, and with BLV sample JN254635 from Mato Grosso State. When compared to GenBank sequences, these samples showed 100% identity with sequence FJ808582, originally from Argentina [24]. Maximum identity was also found between samples BLV 04 (JN254636) from São Paulo State and 13 (JN254640) from Mato Grosso, which were identical to the retrieved sequences from GenBank EF065640, originally described in Costa Rica [25].

A high mutation rate of retroviruses has been reported, linking this to reverse transcriptase due to the high number of replication cycles [26]. The rates of nucleotide mutation of BLV are 0.009% and 0.034% in regions corresponding to the env gene and long terminal repeat (LTR), respectively [27]. However, it was evident in the present work, based on minimal similarities of identity matrices, that both nucleotide (94%) and amino acid (92.4%) present a clear conservation of nucleotide sequences, confirming findings of Portetelle et al. [28] and Felmer et al. [29] that the BLV can conserve its genetic characteristics in different geographical areas for long periods.

**Figure 3:**
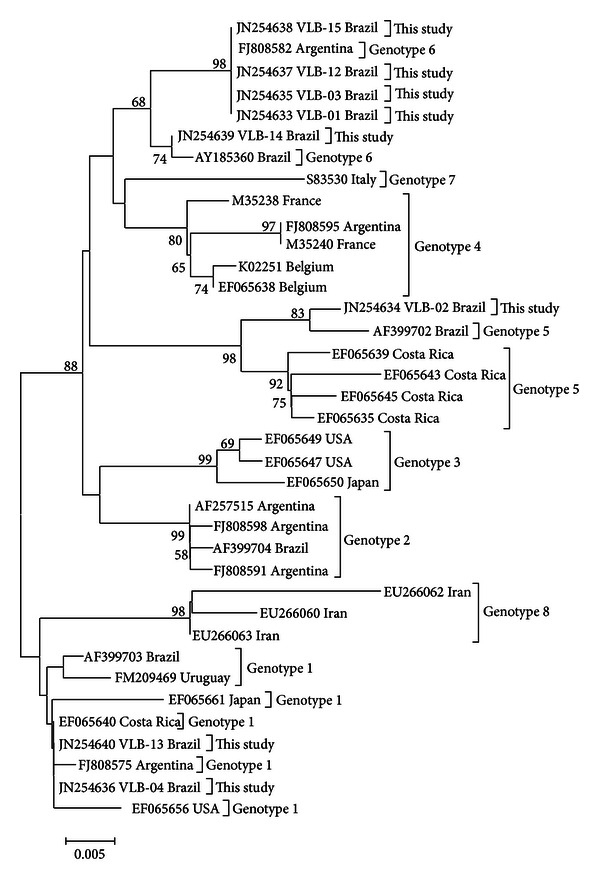
Phylogenetic tree developed by the neighbor-joining method with the substitution pattern *maximum composite likelihood* (Software Mega v. 5). The samples show identification *Accession Number*/country of study/genotype referring to the env gene. The numbers near each node represent the values of 1000 bootstrap replicates, showing only those greater than 50%. The scale represents the number of replacements/sites.

Phylogenetic analysis of eight partial sequences of the env gene (gp51) of BLV showed the presence of three genotypes, they were represented in the phylogenetic tree (Figure 3), and they corresponded to the identity matrix and to the genotypes previously described [24, 30, 31]. Its topology confirmed the separate genotypes reported by these cited authors. Discussions and controversies have been generated concerning the number of genotypes of BLV but have evolved to consensus on the existence of at least seven genotypes, based both on studies using PCR-RFLP and those employing sequencing analyzes [24, 30, 32]. However, evidence for an eighth genotype was reported by Hemmatzadeh [33] and suggested by Matsumura et al. [31], which is why it was considered by this study.

Based on the phylogenetic analysis of the partial region of the env gene, none of the identified genotypes was associated with NPME in cattle with neurological syndrome. There is no currently available information on the pathogenicity of BLV genotypes to different organs, including the bovine CNS. Although the development of a tumoral form of bovine leukosis has been described as dependent on host susceptibility [34, 35], there is currently no evidence for whether some genotypes can influence or induce these neoplasias.

## 4. Conclusions

The results indicate that BLV has no tropism in the CNS, and it cannot be regarded as the primary causative agent of non-purulent meningoencephalitis in cattle. The observed genotypes corroborate previous studies conducted in Brazil. Bovine leukemia virus cannot be excluded from the differential diagnosis of neurological syndrome because, despite being rare, the presence of lymphosarcomas in the CNS has been reported in Brazil.

## Figures and Tables

**Table 1:** Correlation between histopathology of CNS of cattle with neurological syndrome and results of nested-PCR.

Histology (%)	Nested-PCR (%)		Total (%)
	Positive	Negative
NPME	2 (0.7)^a^	72 (26.8)^a^	74 (27.5)
Without alterations	11 (5.6)^a^	184 (68.4)^a^	195 (72.5)
Total (%)	13 (4.8)	256 (95.2)	269 (100)

Same superscript letters between columns are not significantly different at 0.05.

**Table 2:** Sequences with maximum and minimum similarity of nucleotides and of amino acids in the consensus region, when compared with one another and with GenBank sequences.

Similarity	Nucleotide Identity	Amino acid identity
Among the sequences of this study	Maximum (100%) (i) JN254633, JN254635, JN254637 and JN254638 (ii) JN254636 and JN254640	Maximum (100%) (i) JN254633, JN254635, JN254637 and JN254638 (ii) JN254636 and JN254640
	Minimum (95.8%) (i) JN254634 × JN254633, JN254635, JN254637 and JN254638	Minimum (96.5%) (i) JN254634 × JN254633, JN254635, JN254637 and JN254638

Among the sequences of the study before the other retrieved from GenBank	Maximum (100%) (i) JN254633, JN254635, JN254637, JN254638 and FJ808582 (ii) JN254636, JN254640 and EF065640	Maximum (100%) (i) JN254633, JN254635, JN254637, JN254638 and FJ808582 (ii) JN254636, JN254640 and EF065640 (iii) JN254639 and AY185360
	Minimum (94%) (i) JN254634 × EU266062	Minimum (92.4%) (i) EU266062 × JN254634, JN254634, JN254635, JN254638 and JN254638
